# A New Species-Specific Typing Method for Salivarius Group Streptococci Based on the Dephospho-Coenzyme A Kinase (*coaE*) Gene Sequencing

**DOI:** 10.3389/fcimb.2021.685657

**Published:** 2021-08-06

**Authors:** Mohamed M. H. Abdelbary, Gerrit Wilms, Georg Conrads

**Affiliations:** Division of Oral Microbiology and Immunology, Department of Operative and Preventive Dentistry and Periodontology, RWTH Aachen University Hospital, Aachen, Germany

**Keywords:** Streptococcal typing, salivarius group, dephospho-CoA kinase gene, MALDI-TOF MS, streptococcal phylogeny

## Abstract

Viridans streptococci are a group of α-hemolytic streptococcal species. They are mainly commensals, most abundant in the mouth supporting oral health. But they also include important human pathogens such as *Streptococcus pneumoniae*. Identification and molecular typing of viridans group streptococci are challenging, especially for members of the salivarius group. In this study, we developed a single-locus molecular typing method that is able to differentiate among the highly phylogenetically related members of the salivarius group (*S. salivarius*, *S. vestibularis* and *S. thermophilus*) and might support differentiation in other groups as well. This typing approach is based on the amplification and sequence analysis of the housekeeping gene dephospho-coenzyme A kinase (*coaE*), a gene with unrecognized taxonomic potential to date. Here, we analysed *coaE* gene sequences of 154 publicly available genomes and of 30 salivarius group isolates of our own collection that together belong to 20 different gram-positive bacterial (sub) species. Our results revealed that the *coaE* phylogeny distinguished between streptococcal and non-streptococcal genomes and that *coaE* gene sequences were species-specific. In contrast to MALDI-TOF MS performance, the *coaE* typing was able to precisely identify the phylogenetically very closely related members of the salivarius group.

## Introduction

Currently, the genus *Streptococcus* comprises 107 (validly published and correctly named) assigned species (http://www.bacterio.net/streptococcus.html) that diverge between commensals and pathogens to various animals and to humans. Previous studies based on 16S rRNA gene sequence analysis divided most members of the genus *Streptococcus* into six distinct phylogenetic groups, namely the mitis, anginosus, pyogenic, bovis, mutans, and salivarius group. The latter is composed of the oral and intestinal commensals *S. vestibularis* and *S. salivarius* while *S. thermophilus* is essential for manufacturing dairy products and thus frequently consumed in high dosages. On the one hand, *S. thermophilus* and - to a lower extend - *S. salivarius* (strains K12 and M18) are important probiotics. On the other hand, several studies have shown that *S. salivarius* and *S. vestibularis* are opportunistic pathogens and can even be associated with life threating infections such as bacteremia, endocarditis and meningitis (Corredoira et al., 2005; Shewmaker et al., 2010; Srinivasan et al., 2012), the latter occurring mainly in immunocompromised patients.

Previous studies have shown the effectiveness of matrix-assisted laser desorption ionization time-of-flight mass spectrometric (MALDI-TOF MS) in identifying most species among the genus *Streptococcus* (Friedrichs et al., 2007; Lartigue et al., 2009). However, for MALDI-TOF MS the identification and classification of viridans streptococci, in particular those of the salivarius group, remains challenging (Murray, 2010). Furthermore, a total of 11 multilocus sequence typing (MLST) schemes were developed, mainly for pathogenic streptococcal species, such as *S. pneumoniae*, *S. agalactiae*, *S. pyogenes* and *S. bovis*/*equinus* complex. MLST is based on amplification and sequence analysis of seven housekeeping genes. Among the salivarius group species, only *S. thermophilus* has a well-established MLST scheme (Yu et al., 2015). With the introduction of high-throughput sequencing, whole-genome sequence (WGS) analysis has become a valuable typing method for bacterial species due to its high discriminatory power that is able to even differentiate between closely related bacterial isolates. But this method is still expensive and time-consuming.

In this study, we developed a single-locus based typing method that is able to identify and differentiate among the highly phylogenetically related members of the streptococcal salivarius group. This method is based on the amplification and sequence analysis of the housekeeping gene encoding dephospho-coenzyme A kinase (*coaE*). The *coaE* gene was first described in *Escherichia coli* and later detected in several bacterial species and in archaea (Mishra et al., 2001; Shimosaka et al., 2019). The product of dephospho-enzyme A kinase is an essential enzyme responsible for catalyzing the final step (the ATP-dependent phosphorylation of dephospho-CoA) in the coenzyme A (CoA) biosynthesis, which is a fundamental cofactor for several cellular reactions in all living organisms (Spry et al., 2008). Due to its essential role in CoA-metabolism, it was previously suggested as a drug target combating *Mycobacterium tuberculosis* (Ballinger et al., 2019). *CoaE* is informative and has a length of around 600 base pairs, which is optimal for amplification. Previous studies have revealed that bacterial, archaeal and eukaryotic CoaEs are a typical pair of analogous enzymes that are structurally and evolutionarily distinct but convergently evolve to catalyze the similar cellular reaction (Galperin et al., 1998; Omelchenko et al., 2010; Shimosaka et al., 2019). Therefore, the distinct evolutionary dynamics of the coaE gene make it suitable as genetic marker for bacterial molecular typing. In addition, we demonstrate the potential discriminatory power of *coaE* to distinguish among several bacterial species unrelated to the genus *Streptococcus*.

## Materials and Methods

### Primer Design and PCR Amplification

We analyzed *coaE* gene sequences from 154 publicly available genomes of 28 different gram-positive bacterial species that included 134 streptococcal and 20 non-streptococcal genomes (**Table S1**). Four to six genomes for each of the following representative species were downloaded in fasta format from the National Center for Biotechnology Information (NCBI) database: Non-streptococci: *Enterococcus faecalis* (n = 5), *E. faecium* (n = 5), *Listeria monocytogenes* (n = 5), *Staphylococcus aureus (n = 5);* streptococci: *S. agalactiae* (n = 5), *S. anginosus* (n = 5), *S. australis* (n = 4)*, S. constellatus* (n = 4)*, S. cristatus* (n = 5)*, S. equinus* (n = 5), *S. gordonii* (n = 5), *S. infantis* (n = 5), *S. intermedius* (n = 6)*, S. mitis* (n = 5), *S. mutans* (n = 5), *S. oralis* subsp. *dentisani* (frequently abbreviated as “*S. dentisani*”) (n = 5), *S. oralis* subsp. *oralis* (frequently abbreviated as “*S. oralis*”) (n = 5), *S. oralis* subsp. *tigurinus* (frequently abbreviated as “*S. tigurinus*”) (n = 5)*, S. parasanguinis* (n = 5)*, S. pneumoniae* (n = 5), *S. pyogenes* (n = 5), *S. sanguinis* (n = 5), and *S. sobrinus* (n = 5). *S. downei* and *S. peroris* were also included but had only two and one publicly available genome, respectively. As the salivarius group streptococci were the principal target in this study, *S. salivarius*, *S. thermophilus*, and *S. vestibularis* were represented by 15, 15 and 7 genomes (summing up to 37 in total), respectively. All *de novo* assembled genomes were annotated using Prokka pipeline version 1.13.0 (Seemann, 2014). Subsequently, *coaE* genes were localized and extracted from entire genomes GenBank (.gbk) files through Artemis version 18.0.3 (Carver et al., 2005). Based on *coaE* gene sequence alignments from the in total 154 investigated genomes, we targeted suitable conserved regions as primer binding sites specific for salivarius group streptococci. Primer sequences were designed and optimized using Primer3Plus (Untergasser et al., 2012). For the salivarius group streptococci, forward primer *coaE*-SG-fwd 5´-TTAACAGGWGGYATTGCTTCAG-3´ and reverse primer *coaE*-SG-rev 5´-CTTMACCTTCTYCTTCAAATCATC-3´ were designed. PCR and Sanger sequencing were performed as described previously (Conrads et al., 2017) with the following conditions: an initial denaturation step at 94°C for 1 min, followed by 30 cycles (each cycle consisting of 94°C for 30 s, 51°C for 30 s and 72°C for 1 min) terminated by a final extension step at 72°C for 4 min. The primer specificity was carefully ascertained *in silico*; however, one *S. mitis* isolate (OMI-317) was included as a negative control to proof salivarius group primer specificity *in vitro* for every run. PCR products purity and length were confirmed by conventional agarose gel electrophoresis.

### Molecular Analysis of Isolates

To evaluate the use of *coaE* as molecular typing target, we included a collection of 30 salivarius group strains (*S. vestibularis*, n = 8; *S. thermophilus* n = 9, and *S. salivarius* n = 13, labelled “OMI” as from the Division of Oral Microbiology and Immunology strain collection) that were isolated from human (blood n = 15, saliva n = 9, feces n = 3, hip joint puncture n = 1, aortic valve n = 1, clinical data not available n = 1) (for further data see **Table S1**). Isolates were grown overnight on Columbia colistin-nalidixic acid (CNA) agar with 5% (vol/vol) sheep blood (Becton Dickinson, Heidelberg, Germany) at 37°C with an atmosphere of 8% CO_2_. All isolates were pre-identified and pre-classified using MALDI-TOF MS (Biotyper, Bruker Daltonik) according to the manufacturer. For DNA-preparation, an appropriate biomass was collected and re-suspended in 1.5 mL Eppendorf tubes in 1 mL 0.9% sodium chloride (NaCl). Tubes were centrifuged for 1 min at 8,000 rpm and the supernatant was discarded. Remained pellets were treated with a mixture of lysozyme and mutanolysin (LM) and incubated for 30 min at 37°C to disrupt the cell walls. Subsequently, the genomic DNA was extracted using the QIAamp^®^ DNA Mini Kit (Qiagen, USA) according to the manufacturer’s instructions.

### *CoaE* Phylogenetic Tree Reconstruction

A Multi-Fasta file of *coaE* gene sequences was aligned using ClustalW algorithm implemented in MEGAX software version 10.0.5 (Kumar et al., 2018). For determining the best‐fit evolutionary model of nucleotide substitutions, we used the model selection function (*Find Best DNA/Protein Models*) implemented in MEGAX software. A model with the lowest *Bayesian Information Criterion* (BIC) scores was considered to describe the substitution pattern the best, and subsequently, was used for the phylogeny reconstruction. The multiple sequence alignments were used to reconstruct a maximum likelihood (ML) phylogenetic tree with PhyML using default settings and applying complete deletion of gaps/missing data and bootstrap test of 1000 replicates. In addition to the ML method, the neighbor-joining (NJ) method with complete deletion of gaps/missing data and bootstrap test of 1000 replicates that is implemented in MEGAX software (Kumar et al., 2018) was used to investigate the self-reliance of the phylogenetic hypothesis of salivarius group.

## Results

**Figure 1:**
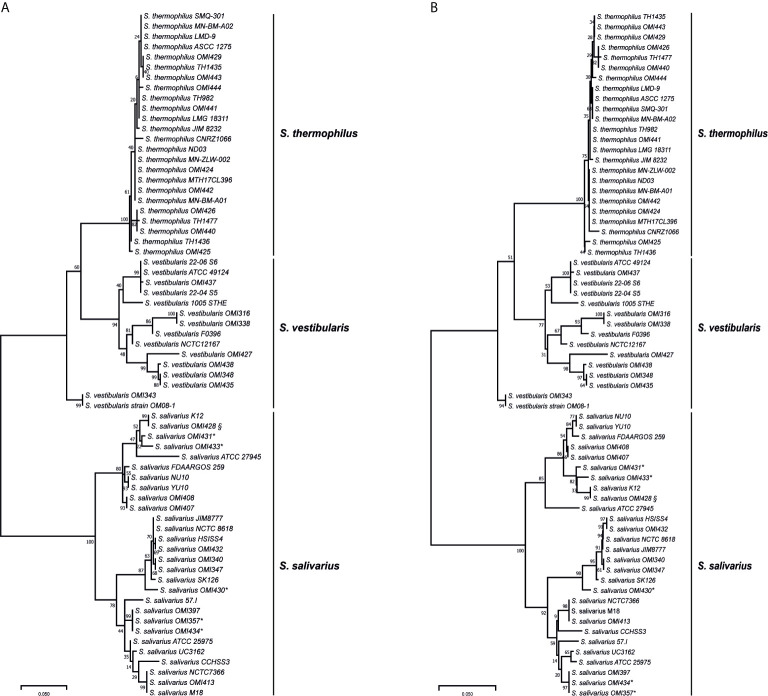
Maximum likelihood **(A)** and neighbor-joining **(B)** phylogenetic trees based on the *coaE* gene sequence of 30 isolates (OMI) and from 37 publicly available salivarius group streptococci genomes imported from NCBI database. * represents isolates that were primarily assigned as “*S. vestibularis*” based on MALDI-TOF MS, while ^§^ shows a subculture of *S. salivarius* strain K12, OMI428, which was misidentified as “*S. vestibularis*” by MALDI-TOF MS. All these isolates were confirmed as *S. salivarius* by applying *coaE* gene typing, suggesting a wrong scoring order by MALDI-TOF MS. Tamura 3-parameter was used as substitution model with a discrete gamma distribution and bootstrap test of 1000 replicates.

Among the salivarius group genomes, the *coaE* gene sequence length was conserved with 594 base pairs (bp). Using this sequence as template, we designed primers targeting the variable region of locus_tag=“SSAL8618_RS03260” in the reference genome of *S. salivarius* strain NCTC 8618 (ATCC 7073) (accession number: NZ_CP009913) at nucleotide positions 13-34 and 538-561 (calculated amplicon length 549 bp), respectively. A total of 30 salivarius group streptococci isolates were characterized using the *coaE* typing approach. This PCR revealed indeed amplicons for all salivarius group isolates, while the laboratory negative-control (*S. mitis* isolate OMI-317) showed no amplification. Sequencing the PCR products of all isolates revealed the expected amplicon size with 549 bp in length. Subsequently, the phylogenetic tree based on the *coaE* gene sequence of these 30 OMI-isolates and of the 37 publicly available salivarius group genomes was constructed using ML method and Tamura 3-parameter substitution model with a discrete gamma distribution as it was shown by model testing to be the best-fit evolutionary model (BIC = 6398.365) (**Figure 1A**). The ML tree showed two main distinct clusters, one representing *S. salivarius* and one consisting of *S. thermophilus* and *S. vestibularis*. *S. vestibularis* had several phylogenetically distinct sub-clusters, differing by 31 single nucleotide polymorphisms (SNPs) in average, that were clearly located closer to the *S. thermophilus* than to the *S. salivarius* clade. This phylogenetic clustering was confirmed by a separate NJ phylogenetic method (**Figure 1B**), which revealed identical two clusters of *S. salivarius*, *S. thermophilus* and *S. vestibularis* with high bootstrap values comparable to those obtained from the ML method (**Figure 1A**).

The isolates OMI357, OMI430, OMI431, OMI433, and OMI434 (marked by * in **Figure 1** and **Table 1**) were primarily assigned as *S. vestibularis* based on MALDI-TOF MS, but showing uncertain results with a score between 2.07 to 2.23 for *S. vestibularis* as first-best match and between 2.04 too 2.15 for *S. salivarius* as second-best match. Clearly, *coaE* gene typing placed these isolates within the *S. salivarius* cluster suggesting a wrong scoring order by MALDI-TOF MS. Another example of misidentification by MALDI-TOF MS was *S. salivarius* strain K12 (subculture OMI428, marked by ^§^ in **Figure 1**) misidentified as *S. vestibularis* (**Table 1**). However, for all previously mentioned isolates, we used in **Figure 1** the right identification species name (*S. salivarius*) according to *coaE* gene typing. The imported *coaE* sequence of strain K12 and the sequencing result of our own K12 subculture (OMI428) were identical, as expected. Taken together, our results show that MALDI-TOF MS failed to assign almost 50% of the investigated *S. salivarius* isolates, while all isolates belonging to *S. thermophilus* and *S. vestibularis* were correctly identified (**Table 1**).

**Table 1:** Comparison between MALDI-TOF MS and *coaE* typing results of the 30 salivarius group streptococcal (OMI) isolates included in this study.

Isolate	First match	Firstmatch score	Second match	Second match score	*coaE *identification
**OMI340**	*S*	2.33	*V*	2.26	*S*
**OMI347**	*S*	2.31	*S*	2.25	*S*
**OMI397**	*S*	2.22	*V*	2.21	*S*
**OMI407**	*S*	2.27	*S*	2.12	*S*
**OMI408**	*S*	2.24	*S*	2.22	*S*
**OMI413**	*S*	2.10	*S*	2.09	*S*
**OMI428§**	*V*	2.18	*V*	2.04	*S*
OMI430*	*V*	2.16	*S*	2.13	*S*
OMI431*	*V*	2.13	*S*	2.09	*S*
**OMI432**	*S*	2.28	*S*	2.10	*S*
OMI433*	*V*	2.16	*S*	2.06	*S*
OMI434*	*V*	2.07	*S*	2.04	*S*
OMI357*	*V*	2.23	*S*	2.15	*S*
**OMI424**	*T*	2.29	*T*	1.93	*T*
**OMI425**	*T*	2.25	*T*	2.13	*T*
**OMI426**	*T*	2.08	*T*	2.01	*T*
**OMI429**	*T*	1.91	*T*	1.90	*T*
**OMI440**	*T*	2.07	*T*	1.96	*T*
**OMI441**	*T*	2.22	*T*	2.11	*T*
**OMI442**	*T*	2.06	*T*	1.88	*T*
**OMI443**	*T*	2.02	*T*	1.99	*T*
**OMI444**	*T*	2.01	*T*	1.94	*T*
**OMI316**	*V*	2.49	*V*	2.40	*V*
**OMI338**	*V*	2.33	*V*	2.32	*V*
**OMI343**	*V*	2.42	*V*	2.36	*V*
**OMI348**	*V*	2.00	*V*	1.98	*V*
**OMI427**	*V*	1.91	*V*	1.90	*V*
**OMI435**	*V*	1.96	*V*	1.86	*V*
**OMI437**	*V*	2.41	*V*	2.28	*V*
**OMI438**	*V*	2.02	*V*	1.92	*V*

S, S. salivarius; V, S. vestibularis; and T, S. thermophilus.

§ represents a subculture of S. salivarius strain K12, and * represents isolates that were primarily assigned as “S. vestibularis” based on MALDI-TOF MS, but coaE gene placed these isolates in the S. salivarius cluster.

In order to investigate the *coaE* gene variability among the 67 salivarius group genomes/isolates, we calculated the mean distance among the different species-specific clades. The results revealed that the *S. salivarius* clade was most divergent from both, *S. vestibularis* and *S. thermophilus* clade by 81 and 93 nucleotide differences, respectively. While *S. vestibularis* and *S. thermophilus* were more closely related and differed by 50 nucleotide substitutions only. Furthermore, we detected a diversity as low as 7 SNPs among isolates of the *S. thermophilus* clade, while the within clade distances of *S. salivarius* and *S. vestibularis* were 27 SNPs and 31 SNPs, respectively.

We elucidated the base composition and genetic variability of the complete *coaE* gene sequence (from ATG to Stop-codon TGA/TAA/TAG, the latter version TAG used in salivarius group), among 154 publicly available genomes that represent 23 streptococcal and four non-streptococcal species. Extracting the *coaE* gene sequences from these 154 genomes revealed a variable and – by excluding *S. infantis* species - specific gene length that ranged between 525 to 630 bp. For example, the largest *coaE* gene was detected in *E. faecium* (630 bp), while the shortest was detected in two out of five genomes of *S. infantis* (525 bp). This heterogeneity in length came along with a large variability in base composition among the five *coaE* gene sequences of *S. infantis*. But with this exception of *S. infantis*, probably indicating either misclassification or subspecies formation, the *coaE* gene had an average of 86% conserved positions on species level and heterogeneity was only found among different species.

**Figure 2:**
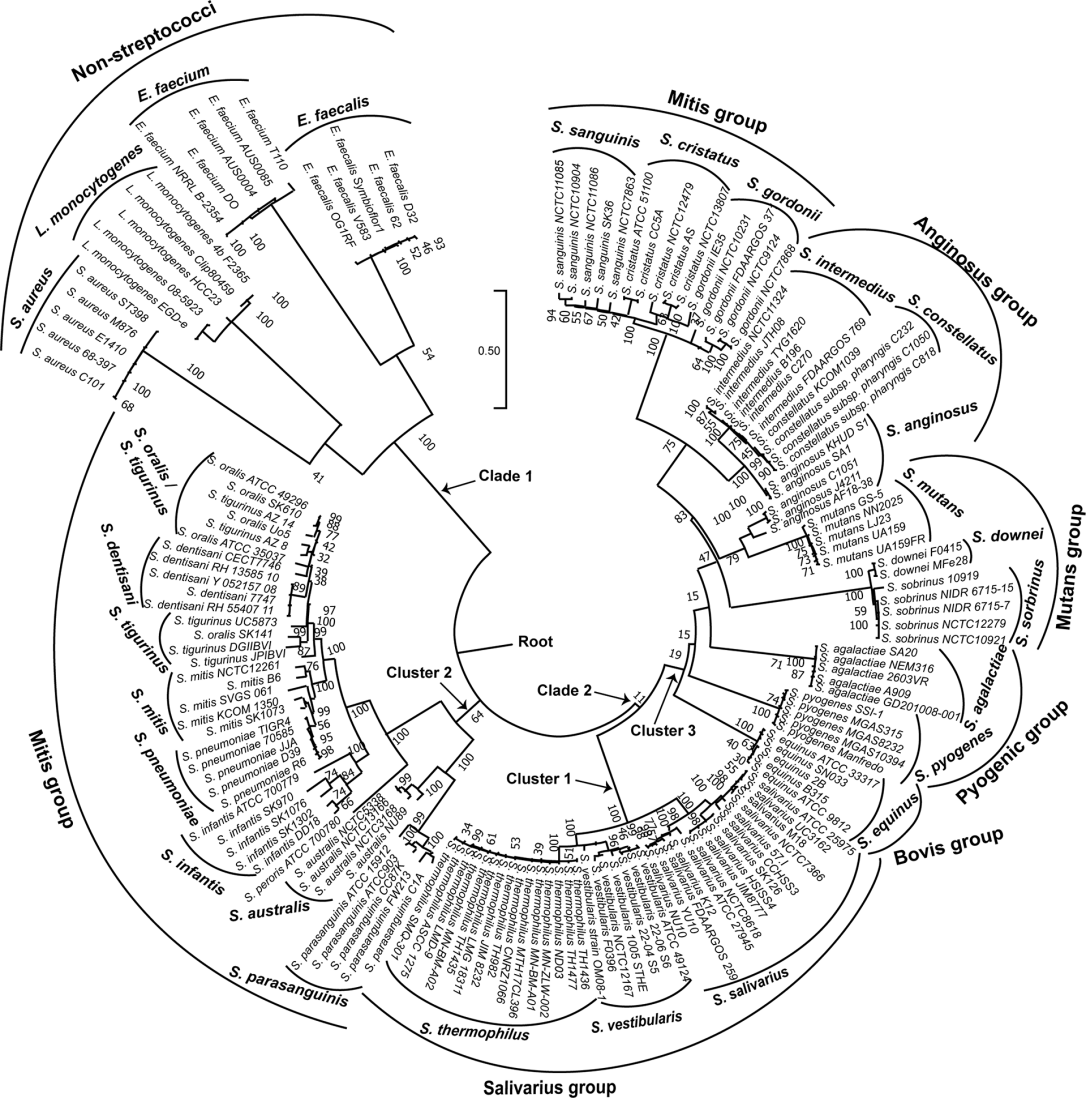
Maximum likelihood phylogenetic tree based on the *coaE* gene sequence of 154 bacterial genomes (134 streptococcal and 20 non-streptococcal genomes). The tree was rooted using the non-streptococcal clade (clade 1) as an outgroup. Tamura 3-parameter was used as substitution model with a discrete gamma distribution of invariable sites and bootstrap test of 1000 replicates.

In order to visualize the relationships among the 154 genomes investigated; we constructed a phylogenetic tree based on the *coaE* gene sequence alignment (**Figure 2**) using ML method and Tamura 3-parameter substitution model with a discrete gamma distribution of invariable sites as it was shown by model testing to be the best-fit evolutionary model (BIC = 36733.568). Interestingly, the ML tree revealed that the 154 genomes made up two main clades with distinct phylogenetic clusters that were strongly associated with the different bacterial species. One distinct phylogenetic clade (clade 1) contained all non-streptococcal genomes consisting of four clusters of *S. aureus*, *L. monocytogenes*, *E. faecalis*, and *E. faecium*. On the other hand, the 134 genomes belonging to the genus *Streptococcus* made up the second distinct clade (clade 2) that was divided into three main clusters. The streptococcal clade differed from the non-streptococcal clade with an average of 304 nucleotide substitutions, while the within clade diversity showed comparable number of nucleotide differences (224 for clade 1 and 222 for clade 2).

**Figure 3:**
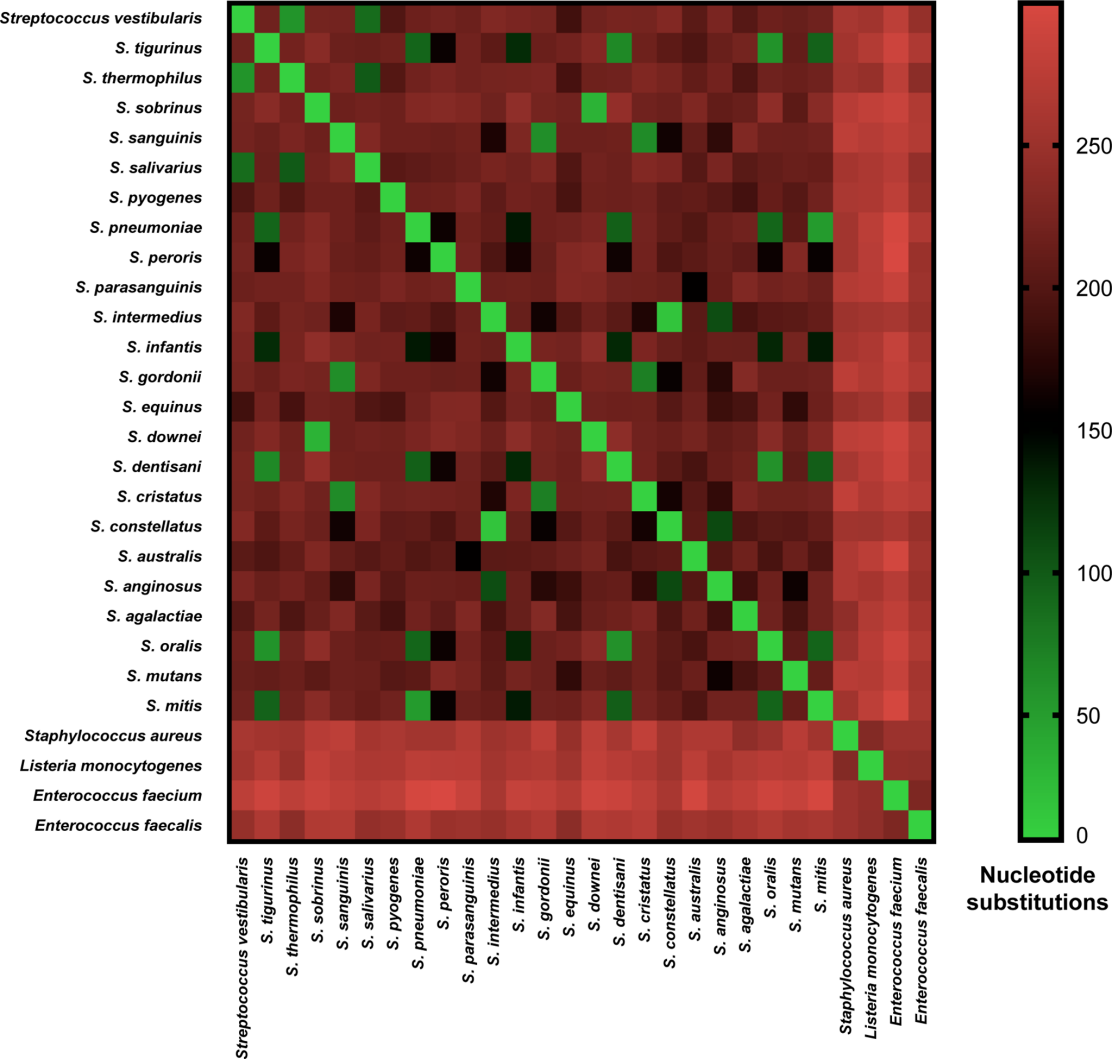
Pairwise nucleotide substitutions heat map. Number of nucleotide substitutions over a 525-630 bp *coaE* gene sequence between 28 different bacterial species as a measure of evolutionary divergence.

Each of the three main streptococcal clusters showed groups and species-specific, but not subspecies-specific sub-clusters (**Figure 2**). For instance, the salivarius group members *S. salivarius*, *S. vestibularis* and *S. thermophilus* together formed a group-specific main cluster (cluster 1) that was divided into individual species-specific subclusters. In addition, most genomes of mitis group members (n = 40) made up cluster 2 including *S. australis*, *S. parasanguinis*, *S. infantis, S. mitis*, *S. pneumoniae*, *S. oralis* subsp. *tigurinus*, *S. oralis* subsp. *dentisani*, *S. oralis* subsp. *oralis*, and *S. peroris*, the latter represented by only one publicly available genome. Similar to cluster 1, species-specific subclusters were also demonstrated in cluster 2. Furthermore, genomes of *S. oralis* subsp. *tigurinus*, *S. oralis* subsp. *dentisani* and *S. oralis* subsp. *oralis* (n = 15) clustered together with limited species-specific resolution, especially for *S. oralis* genomes. For instance, two mixed subclusters (*S. oralis*/*S. tigurinus*) included genomes of *S. oralis* subsp. *tigurinus* and *S. oralis* subsp. *oralis*. In addition*, S. oralis* subsp. *dentisani* made up a distinct subcluster of four genomes, by excluding strain CECT7746, which is an important probiotic that clustered among *S. oralis* subsp. *oralis* and *S. oralis* subsp. *tigurinus* genomes (**Figure 2**). Within cluster 2, *S. mitis* and *S. pneumoniae* were the most closely related species with 44 nucleotide differences only, while the highest number of substitutions (n = 214) was detected between *S. parasanguinis* and *S. peroris* (**Figure 3**).

Cluster 3 harbored the highest number of the investigated streptococcal species (n = 12) that represented four different streptococcal groups (pyogenic, bovis, mutans, anginosus and mitis). Interestingly, the remaining members of mitis group (*S. sanguinis*, *S. gordonii*, and *S. cristatus*), that were included in this analysis, were located in cluster 3 and differed from mitis members in cluster 2 by at least 195 nucleotide substitutions for *S. australis* and *S. cristatus*. The pyogenic group, represented by *S. pyogenes* and *S. agalactiae* did not directly cluster together but were separated by *S. equinus* from the bovis group. However, the pairwise sequence analysis revealed that *coaE* sequences of *S. pyogenes* and *S. agalactiae* differed by 184 nucleotides, while *S. equinus* had 186 and 187 nucleotide differences to *S. agalactiae* and *S. pyogenes*, respectively. In cluster 3, we detected the lowest number of nucleotide substitutions (n = 25) among *S. sobrinus* and *S. downei*, while *S. cristatus* and *S. pyogenes* were the most divergent by 213 nucleotides (**Figure 3**).

## Discussion

Several studies have reported the identification and typing challenges of viridans group streptococci. For instance, the use of MALDI-TOF MS revealed several limitations in the identification of certain species of streptococci such as those within the salivarius group (Murray, 2010). Similarly, the use of DNA-based identification approaches targeting different genes such as 16S rRNA, *groESL*, *rpoB*, *tuf*, and *sodA* were unable to deliver a high discriminatory power for distinguishing between salivarius group species (Simmon et al., 2008; Angeletti et al., 2015).

In this study, we have developed a new typing approach based on the amplification and sequence analysis of *coaE* gene as target marker. In addition, we evaluated the use of *coaE* gene sequences in identifying members of salivarius group streptococci on species level. The clear distinction between salivarius group species is generally difficult, since the 16S rRNA gene of all three species is almost identical (> 99%) (Thompson et al., 2013). Furthermore, a previous study used the *tkt* gene (encoding a transketolase) for the differentiation between *S. salivarius* and *S. vestibularis* (Van den Bogert et al., 2013). However, it was shown that this gene exists in two completely different and variable forms as it can be acquired through horizontal gene transfer (implicating a risk of gene loss or homologous recombination), which makes it unsuitable as typing gene (Delorme et al., 2007). Another molecular typing method based on sequence analysis of the *tuf* gene (encoding the elongation factor Tu) revealed that the salivarius group was monophyletic and *S. salivarius* compared to *S. vestibularis* isolates were 99.1% identical within a *tuf* sequence of 761-bp length (Picard et al., 2004).

Our results revealed that *coaE* gene typing divided the investigated salivarius group genomes into three species-specific clades and was able to identify and differentiate them precisely. Interestingly, MALDI-TOF MS typing of six *S. salivarius* isolates revealed misleading results assigning these isolates as *S. vestibularis* or *S. salivarius* with similar scores. These findings are in agreement with a previous study, which showed that the identification of *S. vestibularis* using MALDI-TOF MS is not reliable (Angeletti et al., 2015). As a clear progress for classification of streptococci, here, *coaE* typing revealed a precise identification of all investigated *S. salivarius* isolates.

However, in contrast to *S. salivarius* and *S. thermophilus* isolates, we detected large *coaE* sequence diversity among the *S. vestibularis* isolates (up to 31 SNPs). In addition, we found that *S. vestibularis* and *S. thermophilus* were closer related to each other (50 nucleotide substitutions) compared to *S. salivarius* (81 and 93 nucleotide substitutions, respectively).

An explanation for the low diversity of 7 SNPs detected within *S. thermophilus* is probably that most *S. thermophilus* public genomes (n = 15) included here represent strains isolated from dairy products. As only a few *S. thermophilus* strains are used as starter culture for the industrial yogurt production and as their identity must be controlled and guaranteed, a low diversity is the consequence (Linares et al., 2016). However, the remaining 9 isolates within *S. thermophilus* clade were retrieved from blood samples, suggesting close genetic relatedness among commensal and pathogenic strains. This implies that the use of further typing methods with higher discriminatory power, such as WGS, should be applied to gain a better understanding of the evolution of *S. thermophilus* pathogenic strains.

The *coaE* typing distinguished between streptococci and non-streptococci species by a mean distance of 304 nucleotide differences, which emphasizes the inter-genus variation of the *coaE* gene. These results show that *coaE* is a suitable target for identifying bacterial species that belong to different genera.

However, the *coaE* gene typing method had a limited resolution to split the *S. oralis* subspecies genomes within the mitis group streptococci. A previous study based on the use of WGS revealed that the three *S. oralis* subspecies form subclusters - within an otherwise coherent phylogenetic clade – with relatively poor separation (Jensen et al., 2016). A former study of our own group subjecting subsp. *S. oralis* and *S. tigurinus* concluded that a clear separation between these subspecies will never be sharp (Conrads et al., 2017) and that linker or hybrid strains do exist. Such hybrids have been described in the mitis group before by Kilian et al. (2008). Taken together, neither *coaE* nor WGS can resolve the principal problem of hybrids.

Previous studies showed that species identification of mitis group streptococci is challenging, with exception of *S. pneumoniae* (Jensen et al., 2016; Velsko et al., 2019). The use of MALDI Biotyper and MALDI-TOF MS revealed misidentifications of non-*S. pneumoniae* mitis strains and some of them were identified as “*S. pneumoniae”* (Kärpänoja et al., 2014; Yahiaoui et al., 2019). The *coaE* typing might help to overcome these false “*S. pneumoniae”* identifications. Our phylogenetic analyses, based on *coaE* gene sequences revealed that the mitis group is made up of different clusters with substantial genetic variety among individual species. These findings are in agreement with a previous study based on WGS suggesting that the mitis group consists of a mixture of genetically distinct and coherent phylogenetic clades (Jensen et al., 2016).

Taken together, the sequence comparisons of *coaE* gene revealed its conservation across the streptococci and non-streptococci species, which directly supports a common ancestor origin of *coaE* followed by speciation of the different bacterial species. In addition, the *coaE* based typing approach evaluated here demonstrated its usefulness for species identification as a standalone method or as an extension of existing concatenated gene sequences for MLST. Furthermore, this method proved to be a precise (100% correct identification of *S. salivarius* isolates) and cost-efficient (single gene) typing strategy for the investigation of salivarius group isolates. This is especially important as members of this group are frequently used as probiotics but can also cause serious clinical conditions, including blood stream infections.

In conclusion, we showed the high value of a *coaE* based typing approach for the precise identification of salivarius group species. The only alternative with a similar or even better discriminatory power is WGS but which is more expensive (∼200 Euros per isolate) and time consuming (24 - 72 h, plus upstream culture 24-96 h and 2 - 4 h downstream data analysis) and requires bioinformatics expertise accessible for only a few laboratories (Deurenberg et al., 2017; Vourli et al., 2021). Therefore, *coaE* analysis is fast and cost-effective as a first-line typing tool for salivarius group streptococci.

## Data Availability Statement

The datasets presented in this study can be found in online repositories. The names of the repository/repositories and accession number(s) can be found below: https://www.ddbj.nig.ac.jp/, accession numbers LC621196 to LC621225.

## Ethics Statement

The oral and fecal *S. salivarius* and *S. vestibularis* isolates (n =11) were obtained from the study velphoro and impact on the oral cavity and gut microbiome. This study was approved by the Ethics Committee (EK No. 270/17) of the Medical Faculty of RWTH Aachen University. All remaining 19 isolates including seven *S. salivarius*, three *S.* vestibularis and all nine *S. thermophilus* were received from the German National Reference Center for Streptococci. For these 19 isolates an ethical approval or patients’ consent was not required since these isolates resulted from routine microbiological diagnostic procedures as requested by the treating physician. All isolates were anonymized and only sample type (saliva, feces or blood) was registered if available.

## Author Contributions

MA comprehended the idea and wrote the manuscript. MA and GC designed the study. GW performed the experiments. GW and MA analysed the data. All authors contributed to the article and approved the submitted version.

## Funding

The study was funded by the START Program of the RWTH Aachen University Hospital (STREPTORANTES #109/19).

## Conflict of Interest

The authors declare that the research was conducted in the absence of any commercial or financial relationships that could be construed as a potential conflict of interest.

## Publisher’s Note

All claims expressed in this article are solely those of the authors and do not necessarily represent those of their affiliated organizations, or those of the publisher, the editors and the reviewers. Any product that may be evaluated in this article, or claim that may be made by its manufacturer, is not guaranteed or endorsed by the publisher.
