# Supplementary material for: A New Species-Specific Typing Method for Salivarius Group Streptococci Based on the Dephospho-Coenzyme A Kinase (coaE) Gene Sequencing
Source: Front Cell Infect Microbiol. 2021 Aug 6;11:685657. doi: 10.3389/fcimb.2021.685657 (PMC8378900; doi:10.3389/fcimb.2021.685657)
Supplement: Supplementary file 1 [file Table_1.docx]

**Table S1**: Bacterial isolates and publicly available genomes included in the present study.

| **Species** | **Isolate** | **Biosample** | **Accession** | **Country** | **Isolation source** | **Institution** | **Reference** |
| --- | --- | --- | --- | --- | --- | --- | --- |
| ***Streptococcus salivarius*** | **NCTC 8618** | SAMN03174835 | NZ_CP009913.1 | United Kingdom | Oral cavity | University of Otago | Direct submission; Heng et al., 2014; |
|  | **JIM8777** | SAMEA2272047 | NC_017595.1 | France | Oral cavity | I.N.R.A, Unite MIG, Domaine de Vilvert | (Guedon et al., 2011) |
|  | **CCHSS3** | SAMEA2272545 | NC_015760.1 | France | Human blood | I.N.R.A, Unite MIG, Domaine de Vilvert | (Delorme et al., 2011b) |
|  | **57.I** | SAMN02603111 | CP002888.1 | Taiwan | Oral cavity | Chang Gung University | (Geng et al., 2011) |
|  | **HSISS4** | SAMN02471219 | NZ_CP013216.1 | The Netherlands | Ileostomy effluent | Top Institute Food and Nutrition TIFN | (Van den Bogert et al., 2013) |
|  | **NU10** | SAMN02720830 | NZ_JJMT00000000.1 | Malaysia | Oral cavity | University of Malaya | (Barbour and Philip, 2014) |
|  | **YU10** | SAMN02720831 | NZ_JJMS00000000.1 | Malaysia | Oral cavity | University of Malaya | (Barbour and Philip, 2014) |
|  | **UC3162** | SAMN03334908 | NZ_JYOY00000000.1 | USA | Oral cavity | University at Buffalo | (Sabharwal et al., 2015) |
|  | **FDAARGOS_259** | SAMN04875585 | NZ_CP020451.2 | USA | Blood | University of Maryland | Direct submission; Goldberg, et al., 2017 |
|  | **ATCC 27945** | SAMN03140396 | NZ_CP015282.1 | USA | Saliva | Illinois Institute of Technology | (Butler et al., 2017) |
|  | **ATCC 25975** | SAMN02401019 | NZ_CP015283.1 | USA | Saliva | Illinois Institute of Technology | (Butler et al., 2017) |
|  | **NCTC7366** | SAMEA3649030 | NZ_LS483366.1 | United Kingdom | NA | The Wellcome Sanger Institute | Direct submission; Doyle et al., 2018 |
|  | **SK126** | SAMN00002153 | NZ_ACLO00000000.1 | USA | Skin | The J. Craig Venter Institute | Direct submission; Sebastian et al., 2009 |
|  | **M18** | SAMN02471912 | NZ_AGBV01000006.1 | United Kingdom | Oral cavity | University of Otago | (Heng et al., 2011) |
|  | **K12 genome** | SAMN02470675 | NZ_ALIF01000007.1 | Switzerland | Saliva child | Nestle Research Center | (Barretto et al., 2012, 12) |
|  | **OMI340** |  | LC621199 | Germany | Saliva | Oral Microbiology and Immunology, RWTH Aachen University Hospital | Present study |
|  | **OMI347** |  | LC621201 | Germany | Faeces | Oral Microbiology and Immunology, RWTH Aachen University Hospital | Present study |
|  | **OMI397** |  | LC621196 | Germany | Saliva | Oral Microbiology and Immunology, RWTH Aachen University Hospital | Present study |
|  | **OMI407** |  | LC621202 | Germany | Saliva | Oral Microbiology and Immunology, RWTH Aachen University Hospital | Present study |
|  | **OMI408** |  | LC621197 | Germany | Faeces | Oral Microbiology and Immunology, RWTH Aachen University Hospital | Present study |
|  | **OMI413** |  | LC621200 | Germany | NA | Oral Microbiology and Immunology, RWTH Aachen University Hospital | Present study |
|  | **OMI428** |  | LC621198 | Germany | K12 lozenges, saliva, healthy individual | Oral Microbiology and Immunology, RWTH Aachen University Hospital | Present study |
|  | **OMI430** |  | LC621206 | Germany | Aortic valve | Oral Microbiology and Immunology, RWTH Aachen University Hospital | Present study |
|  | **OMI431** |  | LC621204 | Germany | Blood | Oral Microbiology and Immunology, RWTH Aachen University Hospital | Present study |
|  | **OMI432** |  | LC621205 | Germany | Blood | Oral Microbiology and Immunology, RWTH Aachen University Hospital | Present study |
|  | **OMI433** |  | LC621207 | Germany | Hip joint puncture | Oral Microbiology and Immunology, RWTH Aachen University Hospital | Present study |
|  | **OMI434** |  | LC621203 | Germany | Blood | Oral Microbiology and Immunology, RWTH Aachen University Hospital | Present study |
|  | **OMI357** |  | LC621211 | Germany | Saliva | Oral Microbiology and Immunology, RWTH Aachen University Hospital | Present study |
| ***Streptococcus thermophilus*** | **TH1435** | SAMN02401100 | NZ_CM002369.1 | Italy | Milk | University of Padua | (Treu et al., 2014c) |
|  | **TH1436** | SAMN02402367 | NZ_CM002370.1 | Italy | Milk | University of Padua | (Treu et al., 2014c) |
|  | **MTH17CL396** | SAMN02437281 | NZ_CM002371.1 | Italy | Cheese | University of Padua | (Treu et al., 2014b, 396) |
|  | **TH982** | SAMN02442657 | NZ_CM003136.1 | Italy | Curd | University of Padua | (Treu et al., 2014a) |
|  | **TH1477** | SAMN02442661 | NZ_CM003135.1 | Italy | Milk | University of Padua | (Treu et al., 2014b, 396) |
|  | **JIM 8232** | SAMEA2272807 | NC_017581.1 | France | Milk | I.N.R.A, Unite MIG, Domaine de Vilvert | (Delorme et al., 2011a) |
|  | **LMG 18311** | SAMN02604079 | NC_006448.1 | France | Yogurt | Institut National de la Recherche Agronomique | (Bolotin et al., 2004) |
|  | **CNRZ1066** | SAMN02603344 | NC_006449.1 | France | Yogurt | Institut National de la Recherche Agronomique | (Bolotin et al., 2004) |
|  | **LMD-9** | SAMN02598313 | NC_008532.1 | USA | Fermented dairy food | US DOE Joint Genome Institute | (Makarova et al., 2006) |
|  | **ND03** | SAMN02603938 | NC_017563.1 | China | Fermented yak milk | State Key Laboratory of Food Science and Technology | (Sun et al., 2011, 03) |
|  | **MN-ZLW-002** | SAMN02603848 | NC_017927.1 | China | Fermented Chinese dairy products | Research and Development System, Inner Mongolia Mengniu Dairy Group Co. Ltd. | (Kang et al., 2012) |
|  | **ASCC 1275** | SAMN02770279 | NZ_CP006819.1 | China | Dairy starter bacterium | The University of Hong Kong | (Wu et al., 2015) |
|  | **SMQ-301** | SAMN02850632 | NZ_CP011217.1 | Canada | Dairy starter bacterium | Departement de Biochimie, de Microbiologie et de Bio-Informatique | Direct submission; Labrie et al., 2015 |
|  | **MN-BM-A02** | SAMN03325854 | NZ_CP010999.1 | China | Yogurt | Research and Development System, Inner Mongolia Mengniu Dairy Group Co. Ltd. | (Shi et al., 2015) |
|  | **MN-BM-A01** | SAMN04002535 | NZ_CP012588.1 | China | Fermented Chinese dairy products | Research and Development System, Inner Mongolia Mengniu Dairy Group Co. Ltd. | Direct submission; Bai et al., 2015 |
|  | **OMI424** |  | LC621217 | Germany | Blood | Oral Microbiology and Immunology, RWTH Aachen University Hospital | Present study |
|  | **OMI425** |  | LC621218 | Germany | Blood | Oral Microbiology and Immunology, RWTH Aachen University Hospital | Present study |
|  | **OMI426** |  | LC621219 | Germany | Blood | Oral Microbiology and Immunology, RWTH Aachen University Hospital | Present study |
|  | **OMI429** |  | LC621216 | Germany | Blood | Oral Microbiology and Immunology, RWTH Aachen University Hospital | Present study |
|  | **OMI440** |  | LC621221 | Germany | Blood | Oral Microbiology and Immunology, RWTH Aachen University Hospital | Present study |
|  | **OMI441** |  | LC621223 | Germany | Blood | Oral Microbiology and Immunology, RWTH Aachen University Hospital | Present study |
|  | **OMI442** |  | LC621224 | Germany | Blood | Oral Microbiology and Immunology, RWTH Aachen University Hospital | Present study |
|  | **OMI443** |  | LC621222 | Germany | Blood | Oral Microbiology and Immunology, RWTH Aachen University Hospital | Present study |
|  | **OMI444** |  | LC621225 | Germany | Blood | Oral Microbiology and Immunology, RWTH Aachen University Hospital | Present study |
| ***Streptococcus vestibularis*** | **ATCC 49124** | SAMN00253297 | NZ_AEVI00000000.1 | USA | Oral cavity | Baylor College of Medicine | Direct submission; Muzny et al., 2011 |
|  | **F0396** | SAMN00115117 | NZ_AEKO00000000.1 | USA | Oral | The J. Craig Venter Institute | Direct submission; Durkin et al., 2010 |
|  | **1005_STHE** | SAMN03196967 | NZ_JWGP00000000.1 | USA | Broncho alveolar lavage | University of Washington | Direct submission; Roach, 2015 |
|  | **22-04 S5** | SAMN04931587 | NZ_LXZW00000000.1 | Brazil | Oral cavity | University of Campinas | (Palma et al., 2016) |
|  | **22-06 S6** | SAMN04931594 | NZ_LXZX00000000.1 | Brazil | Oral cavity | University of Campinas | (Palma et al., 2016) |
|  | **NCTC12167** | SAMEA3594358 | NZ_LR134275.1 | United Kingdom | Oral cavity | The Wellcome Sanger Institute | Direct submission; Pathogen Informatics, 2016 |
|  | **OM08-1** | SAMN09736843 | NZ_QSTK00000000.1 | China | Faeces | BGI-CNGB | Direct submission; Zou et al., 2018 |
|  | **OMI316** |  | LC621208 | Germany | Saliva | Oral Microbiology and Immunology, RWTH Aachen University Hospital | Present study |
|  | **OMI338** |  | LC621209 | Germany | Saliva | Oral Microbiology and Immunology, RWTH Aachen University Hospital | Present study |
|  | **OMI343** |  | LC621212 | Germany | Saliva | Oral Microbiology and Immunology, RWTH Aachen University Hospital | Present study |
|  | **OMI348** |  | LC621210 | Germany | Faeces | Oral Microbiology and Immunology, RWTH Aachen University Hospital | Present study |
|  | **OMI427** |  | LC621220 | Germany | Saliva | Oral Microbiology and Immunology, RWTH Aachen University Hospital | Present study |
|  | **OMI435** |  | LC621214 | Germany | Blood | Oral Microbiology and Immunology, RWTH Aachen University Hospital | Present study |
|  | **OMI437** |  | LC621215 | Germany | Blood | Oral Microbiology and Immunology, RWTH Aachen University Hospital | Present study |
|  | **OMI438** |  | LC621213 | Germany | Blood | Oral Microbiology and Immunology, RWTH Aachen University Hospital | Present study |
| ***Streptococcus agalactiae*** | **2603V/R** | SAMN02604013 | NC_004116.1 | NA | NA | The Institute for Genomic Research | (Tettelin et al., 2002) |
|  | **A909** | SAMN02604011 | NC_007432.1 | USA | NA | The Institute for Genomic Research | (Tettelin et al., 2005) |
|  | **NEM316** | SAMEA3138330 | NC_004368.1 | France | Blood septicaemia | Institut Pasteur | (Glaser et al., 2002) |
|  | **GD201008-001** | SAMN02603155 | NC_018646.1 | China | Tilapia causing meningoencephalitis | Nanjing Agricultural University | (Liu et al., 2012) |
|  | **SA20** | SAMN02603506 | NC_019048.2 | Brazil | Kidney of infected fish | Universidade Federal de Minas | (Pereira et al., 2013, 20–06) |
| ***Streptococcus pyogenes*** | **MGAS8232** | SAMN02603495 | NC_003485.1 | USA | Respiratory system | Rocky Mountain Laboratories | (Smoot et al., 2002) |
|  | **MGAS315** | SAMN02603496 | NC_004070.1 | USA | NA | Rocky Mountain Laboratories | (Beres et al., 2002) |
|  | **Manfredo** | SAMEA1705956 | NC_009332.1 | USA | NA | The Wellcome Sanger Institute | (Holden et al., 2007) |
|  | **SSI-1** | SAMD00061102 | NC_004606.1 | Japan | NA | University of Tokyo | (Nakagawa, 2003) |
|  | **MGAS10394** | SAMN02603497 | NC_006086.1 | USA | Nasopharynx | Rocky Mountain Laboratories | (Banks et al., 2004) |
| ***Streptococcus mutans*** | **UA159** | SAMN02604090 | NC_004350.2 | USA | Oral | University of Oklahoma Health Sciences Center | (Ajdic et al., 2002) |
|  | **NN2025** | SAMD00060939 | NC_013928.1 | Japan | Oral | Ichiro Nakagawa Tokyo Medical and Dental University | (Maruyama et al., 2009) |
|  | **GS-5** | SAMN02603489 | NC_018089.1 | USA | Carious lesions | University of Kansas Medical Center | (Biswas and Biswas, 2012) |
|  | **LJ23** | SAMD00061051 | NC_017768.1 | Japan | Oral cavity | Fumito Maruyama Tokyo Medical and Dental University | (Aikawa et al., 2012) |
|  | **UA159-FR** | SAMN03278343 | NZ_CP007016.1 | China | NA | Jilin University | Direct submission; Zhang et al., 2013 |
| ***Streptococcus parasanguinis*** | **ATCC 15912** | SAMN00113608 | NC_015678.1 | USA | Oral cavity | Baylor College of Medicine | Direct submission; Muzny et al., 2011 |
|  | **FW213** | SAMN02603112 | NC_017905.1 | China | Dental plaque | Chang Gung University | (Geng et al., 2012) |
|  | **ATCC 903** | SAMN00253299 | NZ_AEVE00000000.1 | USA | Upper respiratory tract | Baylor College of Medicine | Direct submission; Muzny et al., 2011 |
|  | **CC87K** | SAMN01162070 | NZ_AZJD00000000.1 | USA | NA | Broad Institute | Direct submission; Earl et al., 2013 |
|  | **C1A** | SAMN02768894 | NZ_JMRV00000000.1 | Malaysia | Sputum | University of Malaya | (Chan et al., 2015) |
| ***Streptococcus australis*** | **NCTC3168** | SAMEA3649029 | LR134285 | United Kingdom | NA | The Wellcome Sanger Institute | Direct submission; Doyle et al., 2018 |
|  | **NCTC5338** | SAMEA3614271 | LR594040 | United Kingdom | NA | The Wellcome Sanger Institute | Direct submission; Doyle et al., 2018 |
|  | **NCTC13166** | SAMEA103899862 | LS483444 | United Kingdom | NA | The Wellcome Sanger Institute | Direct submission; Doyle et al., 2018 |
|  | **NU89** | SAMN09060970 | NZ_QFBE01000001 | United Kingdom | otitis media | Oral Biology, Newcastle University | Direct submission; Wayes et al., 2018 |
| ***Streptococcus equinus*** | **ATCC 9812** | SAMN00217012 | NZ_AEVB00000000.1 | USA | Gastrointestinal tract | Baylor College of Medicine | Direct submission; Muzny et al., 2010 |
|  | **B315** | SAMN02440831 | NZ_AUJD00000000.1 | Canada | NA | DOE Joint Genome Institute | Direct submission; Kelly et al., 2013 |
|  | **2B** | SAMN02841195 | NZ_JNKQ00000000.1 | Canada | NA | DOE Joint Genome Institute | Direct submission; Kelly et al., 2014 |
|  | **SN033** | SAMN02440584 | NZ_ATWZ00000000.1 | Canada | NA | DOE Joint Genome Institute | Direct submission; Kelly et al., 2013 |
|  | **ATCC 33317** | SAMN01983951 | NZ_AUZG00000000.1 | USA | Cow dung | FDA/CVM | (Benahmed et al., 2014) |
| ***Streptococcus anginosus*** | **C1051** | SAMN02603662 | NC_022244.1 | Canada | Blood sepsis | Public Health Agency of Canada, National Microbiology Laboratory | (Olson et al., 2013) |
|  | **SA1** | SAMN05771128 | NZ_CP007573.1 | USA | Catheterized urine | Centers for Disease Control and Prevention | (Srinivasan et al., 2014) |
|  | **J4211** | SAMN03577772 | NZ_CP012805.1 | USA | NA | University of Oklahoma Health Sciences Center | (Rahman et al., 2015) |
|  | **AF18-38** | SAMN09734365 | NZ_QRWZ00000000.1 | China | Faeces | BGI-CNGB | (Zou et al., 2019) |
|  | **KHUD_S1** | SAMN10458461 | NZ_RSCG00000000.1 | South Korea | Oral cavity | Kyung Hee University | Direct submission; Lee et al., 2018 |
| ***Streptococcus intermedius*** | **TYG1620** | SAMD00031898 | AP014880 | NA | Human Brain Abscess | Pathogen Genomics Center, National Institute of Infectious Diseases | (Hasegawa et al., 2017) |
|  | **NCTC11324** | SAMEA4012327 | LS483436 | United Kingdom | NA | The Wellcome Sanger Institute | Direct submission; Doyle et al., 2018 |
|  | **FDAARGOS_769** | SAMN11056484 | CP053999 | USA | NA | Center for Devices and Radiological Health | Direct submission; M-Clelandal., 2020 |
|  | **JTH08** | SAMD00060960 | AP010969 | Japan | NA | Tadashi Baba Juntendo University | Direct submission; Kikuchi et al., 2009 |
|  | **C270** | SAMN02603658 | CP003858 | Canada | Broncho-pulmonary, | Public Health Agency of Canada | (Olson et al., 2013) |
|  | **B196** | SAMN02603659 | CP003857 | Canada | Broncho-pulmonary, | Public Health Agency of Canada | (Olson et al., 2013) |
| ***Streptococcus constellatus*** | **C818** | SAMN02603660 | CP003840 | Canada | Recurrent broncho-pulmonary | Public Health Agency of Canada | (Olson et al., 2013) |
|  | **C1050** | SAMN02603661 | CP003859 | Canada | Blood sepsis | Public Health Agency of Canada | (Olson et al., 2013) |
|  | **C232** | SAMN02603657 | CP003800 | Canada | Recurrent broncho-pulmonary | Public Health Agency of Canada | (Olson et al., 2013) |
|  | **KCOM 1039** | SAMN09781071 | NZ_QWKV01000001 | Korea | NA | Korean Collection for Oral Microbiology | Direct submission; Kook et al., 2018 |
| ***Streptococcus infantis*** | **ATCC 700779** | SAMN00216972 | NZ_AEVD00000000.1 | USA | Airways | Baylor College of Medicine | Direct submission; Muzny et al., 2010 |
|  | **SK970** | SAMN00621709 | NZ_AFUT00000000.1 | USA | Blood | The J. Craig Venter Institute | Direct submission; Harkins et al., 2011 |
|  | **SK1076** | SAMN00621697 | NZ_AFNN00000000.1 | USA | Bood | The J. Craig Venter Institute | Direct submission; Durkin et al., 2011 |
|  | **DD18** | SAMN04325030 | NZ_CM003841.1 | Germany | Oral cavity | University of Kaiserslautern | (Denapaite et al., 2016) |
|  | **SK1302** | SAMN02435821 | NZ_AEDY00000000.1 | USA | Oral cavity | University of Maryland | Direct submission; Daugherty et al., 2010 |
| ***Streptococcus mitis*** | **B6** | SAMEA3138244 | NC_013853.1 | Germany | Oral | University of Kaiserslautern | (Denapaite et al., 2010) |
|  | **NCTC 12261** | SAMN02435817 | NZ_CP028414.1 | USA | Oral | University of Maryland School of Medicine | Direct submission; Nadendla et al., 2010 |
|  | **KCOM 1350 = ChDC B183** | SAMN03263069 | NZ_CP012646.1 | Korea | Oral cavity | Korean Collection for Oral Microbiology and Department of Oral Biochemistry | Direct submission; Kook et al., 2015 |
|  | **SVGS_061** | SAMN04461788 | NZ_CP014326.1 | USA | Blood | Diagnostic Genetics SHP | (Petrosyan et al., 2016) |
|  | **SK1073** | SAMN00621702 | NZ_AFQT00000000.1 | USA | Blood | The J. Craig Venter Institute | Direct submission; Durkin et al., 2011 |
| ***Streptococcus pneumoniae*** | **R6** | SAMN02603218 | NC_003098.1 | USA | Laboratory strain | Infectious Diseases Research, Eli Lilly and Company | (Hoskins et al., 2001, 6) |
|  | **TIGR4; ATCC BAA-334** | SAMN02604002 | NC_003028.3 | USA | Blood | University of Maryland School of Medicine | (Tettelin, 2001) |
|  | **D39** | SAMN02604051 | NC_008533.2 | USA | NA | The Institute for Genomic Research | (Lanie et al., 2007) |
|  | **70585** | SAMN02603446 | NC_012468.1 | USA | NA | The J. Craig Venter Institute | Direct submission; Hotopp et al., 2007 |
|  | **JJA** | SAMN02603444 | NC_012466.1 | USA | NA | The J. Craig Venter Institute | Direct submission; Hotopp et al., 2007 |
| ***Streptococcus oralis*** | **Uo5** | SAMEA2272261 | NC_015291.1 | Hungary | NA | University of Kaiserslautern | (Reichmann et al., 2011, 5) |
|  | **ATCC 35037** | SAMN00120580 | NZ_ADMV00000000.1 | USA | NA | Baylor College of Medicine | Direct submission; Muzny et al., 2009 |
|  | **ATCC 49296** | SAMN00260261 | NZ_AEPO00000000.1 | USA | Oral cavity | Baylor College of Medicine | Direct submission; Muzny et al., 2010 |
|  | **SK141** | SAMN02836942 | NZ_JPGA00000000.1 | USA | Dental biofilm | University of Maryland School of Medicine | (Kilian et al., 2014) |
|  | **SK610** | SAMN00761855 | NZ_AJKQ00000000.1 | USA | Throat | The J. Craig Venter Institute | Direct submission; Durkin et al., 2012 |
| ***Streptococcus tigurinus*** | **UC5873** | SAMN03334904 | NZ_JYGU00000000.1 | USA | Oral cavity | University at Buffalo | (Sabharwal et al., 2015) |
|  | **JPIBVI** | SAMN03480682 | NZ_LAWI00000000.1 | USA | Dental plaque | University of Malaya | (Zheng et al., 2016) |
|  | **DGIIBVI** | SAMN03480631 | NZ_LAWC00000000.1 | USA | Dental plaque | University of Malaya | (Zheng et al., 2016) |
|  | **AZ_14** | SAMN04283922 | NZ_LNVG00000000.1 | Switzerland | Blood | University Hospital of Geneva | (Diene et al., 2016) |
|  | **AZ_8** | SAMN04283921 | NZ_LNVF00000000.1 | Switzerland | Blood | University Hospital of Geneva | (Diene et al., 2016) |
| ***Streptococcus dentisani*** | **RH_55407_11** | SAMN06705609 | NZ_NCUZ00000000.1 | Denmark | Blood | Slagelse Hospital | (Rasmussen et al., 2016) |
|  | **RH_13585_10** | SAMN06705608 | NZ_NCVA00000000.1 | Denmark | Blood | Slagelse Hospital | (Rasmussen et al., 2016) |
|  | **Y_052157_08** | SAMN06705607 | NZ_NCVB00000000.1 | Denmark | Blood | Slagelse Hospital | (Rasmussen et al., 2016) |
|  | **7747** | SAMEA2272194 | NZ_CAUK00000000.1 | Spain | Tooth surface | Advanced Centre for Public Health Research | (Camelo-Castillo et al., 2014) |
|  | **CECT 7746** | SAMEA2272693 | NZ_CAUJ00000000.1 | Spain | Tooth surface | Advanced Centre for Public Health Research | (Camelo-Castillo et al., 2014) |
| ***Streptococcus peroris*** | **ATCC 700780** | SAMN00253298 | NZ_AEVF00000000.1 | USA | Oral cavity | Baylor College of Medicine | Direct submission; Muzny et al., 2010 |
| ***Streptococcus sobrinus*** | **NIDR 6715-7** | SAMN09232464 | CP029560 | USA | NA | University of Illinois at Urbana-Champaign | Direct Submission; Sales,M et al., 2018 |
|  | **NIDR 6715-15** | SAMN09232465 | CP029559 | USA | NA | University of Illinois at Urbana-Champaign | Direct Submission; Sales,M et al., 2018 |
|  | **10919** | SAMN09214223 | CP029491 | USA | NA | University of Illinois at Urbana-Champaign | Direct Submission; Sales,M et al., 2018 |
|  | **NCTC12279** | SAMEA3729942 | LS483378 | United Kingdom | NA | The Wellcome Sanger Institute | Direct submission; Doyle et al., 2018 |
|  | **NCTC10921** | SAMEA3729941 | LS483381 | United Kingdom | NA | The Wellcome Sanger Institute | Direct submission; Doyle et al., 2018 |
| ***Streptococcus sanguinis*** | **NCTC11086** | SAMEA3649033 | LS483364 | United Kingdom | NA | The Wellcome Sanger Institute | Direct submission; Doyle et al., 2018 |
|  | **NCTC11085** | SAMEA3632064 | LS483346 | United Kingdom | NA | The Wellcome Sanger Institute | Direct submission; Doyle et al., 2018 |
|  | **NCTC 10904** | SAMEA3307890 | LR134002 | United Kingdom | NA | The Wellcome Sanger Institute | Direct submission; Doyle et al., 2018 |
|  | **NCTC7863** | SAMEA3672886 | LS483385 | United Kingdom | NA | The Wellcome Sanger Institute | Direct submission; Doyle et al., 2018 |
|  | **SK36** | SAMN02604299 | CP000387 | USA | Dental plaque | Virginia Commonwealth University | (Xu et al., 2007) |
| ***Streptococcus cristatus*** | **AS** | SAMN02603400 | CP004409 | China | NA | Institute of Microbiology, Chinese Academy of Sciences | (Tong et al., 2013) |
|  | **ATCC 51100** | SAMN14365916 | CP050133 | USA | NA | Vaccine and Infectious Disease, Fred Hutchinson Cancer Research | Direct submission; Johnston et al., 2020 |
|  | **NCTC12479** | SAMEA3649037 | LS483383 | United Kingdom | NA | The Wellcome Sanger Institute | Direct submission; Doyle et al., 2018 |
|  | **NCTC13807** | SAMEA44545168 | LS483471 | United Kingdom | NA | The Wellcome Sanger Institute | Direct submission; Doyle et al., 2018 |
|  | **CC5A** | SAMN03334893 | NZ_JYGJ01000004 | NA | Oral cavity | University at Buffalo | (Sabharwal et al., 2015) |
| ***Streptococcus downei*** | **F0415** | SAMN00115114 | NZ_AEKN01000003 | USA | NA | The J. Craig Venter Institute | Direct submission; Durkin et al., 2010 |
|  | **MFe28** | SAMEA4534871 | NZ_UHFA01000002 | United Kingdom | NA | The Wellcome Sanger Institute | Direct submission; Doyle et al., 2018 |
| ***Streptococcus gordonii*** | **NCTC9124** | SAMEA3643307 | LR594041 | United Kingdom | NA | The Wellcome Sanger Institute | Direct submission; 2019 |
|  | **NCTC7868** | SAMEA3649040 | LR134291 | United Kingdom | NA | The Wellcome Sanger Institute | Direct submission; 2018 |
|  | **NCTC10231** | SAMEA3662936 | LR594049 | United Kingdom | NA | The Wellcome Sanger Institute | Direct submission; 2019 |
|  | **FDAARGOS_371** | SAMN07312415 | CP023511 | USA | NA | Center for Devices and Radiological Health | Direct submission; Campos et al., 2017 |
|  | **IE35** | SAMN03445771 | CP017295 | India | NA | Medical Microbiology, University of Madras | Direct submission; Naveen Kumar et al., 2017 |
| ***Staphylococcus aureus*** | **type strain:ST398** | SAMEA2272644 | NC_017333.1 | The Netherlands | Blood | UMC Utrecht | (Schijffelen et al., 2010, 398) |
|  | **68-397** | SAMN02595314 | NZ_ACJT00000000.1 | USA | Nasal | Broad Institute of MIT and Harvard | Direct submission; Feldgarden et al., 2009 |
|  | **E1410** | SAMN02595315 | NZ_ACJU00000000.1 | USA | NA | Broad Institute of MIT and Harvard | Direct submission; Feldgarden et al., 2009 |
|  | **M876** | SAMN02595316 | NZ_ACJV00000000.1 | USA | NA | Broad Institute of MIT and Harvard | Direct submission; Feldgarden et al., 2009 |
|  | **C101** | SAMN02595338 | NZ_ACSP00000000.1 | USA | NA | Broad Institute of MIT and Harvard | Direct submission; Feldgarden et al., 2009 |
| ***Enterococcus faecium*** | **DO** | SAMN00002237 | NC_017960.1 | USA | Blood | Baylor College of Medicine | (Qin et al., 2012, 16) |
|  | **Aus0004** | SAMN02604218 | NC_017022.1 | Australia | Blood | University of Melbourne | (Lam et al., 2012) |
|  | **NRRL B-2354** | SAMN02604149 | NC_020207.1 | USA | Milk and dairy utensils | University of California | (Kopit et al., 2014) |
|  | **AUS0085** | SAMN02604219 | NC_021994.1 | Australia | Blood | University of Melbourne | (Lam et al., 2013, 203) |
|  | **T110** | SAMN02194078 | NZ_CP006030.1 | India | NA | SRM University | (Natarajan and Parani, 2015) |
| ***Enterococcus faecalis*** | **V583** | SAMN02603978 | NC_004668.1 | USA | Blood | The Institute for Genomic Research | (Paulsen, 2003) |
|  | **OG1RF** | SAMN02603002 | NC_017316.1 | USA | Blood | Baylor College of Medicine | (Bourgogne et al., 2008) |
|  | **62** | SAMN02603509 | CP002491.1 | Norway | Faeces | Norwegian University of Life Sciences | (Brede et al., 2011) |
|  | **D32** | SAMN02603765 | NC_018221.1 | Germany | Faeces | Robert Koch Institute | (Zischka et al., 2012) |
|  | **Symbioflor 1** | SAMEA2272087 | NC_019770.1 | Germany | NA | Justus-Liebig-Universitaet Giessen | (Fritzenwanker et al., 2013) |
| ***Listeria monocytogenes*** | **EGD-e** | SAMEA3138329 | NC_003210.1 | France | NA | Institut Pasteur | (Doumith et al., 2004) |
|  | **4b F2365** | SAMN02603980 | NC_002973.6 | USA | Cheese | The Institute for Genomic Research | (Nelson, 2004) |
|  | **HCC23** | SAMN02603154 | NC_011660.1 | USA | Catfish | Mississippi State University | (Steele et al., 2011) |
|  | **08-5923** | SAMN02603721 | NC_013768.1 | Canada | Blood | Public Health Agency of Canada | (Gilmour et al., 2010) |
|  | **Clip80459** | SAMEA2272134 | NC_012488.1 | France | NA | Institut Pasteur | Direct submission; Rusniok, 2008 |
|  |  |  |  |  |  |  |  |

NA: not available

**References:**

Aikawa, C., Furukawa, N., Watanabe, T., Minegishi, K., Furukawa, A., Eishi, Y., et al. (2012). Complete Genome Sequence of the Serotype k Streptococcus mutans Strain LJ23. *Journal of Bacteriology* 194, 2754–2755. doi:10.1128/JB.00350-12.

Ajdic, D., McShan, W. M., McLaughlin, R. E., Savic, G., Chang, J., Carson, M. B., et al. (2002). Genome sequence of Streptococcus mutans UA159, a cariogenic dental pathogen. *Proceedings of the National Academy of Sciences* 99, 14434–14439. doi:10.1073/pnas.172501299.

Banks, D. J., Porcella, S. F., Barbian, K. D., Beres, S. B., Philips, L. E., Voyich, J. M., et al. (2004). Progress toward Characterization of the Group A *Streptococcus* Metagenome: Complete Genome Sequence of a Macrolide‐Resistant Serotype M6 Strain. *J INFECT DIS* 190, 727–738. doi:10.1086/422697.

Barbour, A., and Philip, K. (2014). Variable Characteristics of Bacteriocin-Producing Streptococcus salivarius Strains Isolated from Malaysian Subjects. *PLoS ONE* 9, e100541. doi:10.1371/journal.pone.0100541.

Barretto, C., Alvarez-Martin, P., Foata, F., Renault, P., and Berger, B. (2012). Genome Sequence of the Lantibiotic Bacteriocin Producer Streptococcus salivarius Strain K12. *Journal of Bacteriology* 194, 5959–5960. doi:10.1128/JB.01268-12.

Benahmed, F. H., Gopinath, G. R., Harbottle, H., Cotta, M. A., Luo, Y., Henderson, C., et al. (2014). Draft Genome Sequences of Streptococcus bovis Strains ATCC 33317 and JB1. *Genome Announcements* 2, e01012-14, 2/5/e01012-14. doi:10.1128/genomeA.01012-14.

Beres, S. B., Sylva, G. L., Barbian, K. D., Lei, B., Hoff, J. S., Mammarella, N. D., et al. (2002). Genome sequence of a serotype M3 strain of group A Streptococcus: Phage-encoded toxins, the high-virulence phenotype, and clone emergence. *Proceedings of the National Academy of Sciences* 99, 10078–10083. doi:10.1073/pnas.152298499.

Biswas, S., and Biswas, I. (2012). Complete Genome Sequence of Streptococcus mutans GS-5, a Serotype c Strain. *Journal of Bacteriology* 194, 4787–4788. doi:10.1128/JB.01106-12.

Bolotin, A., Quinquis, B., Renault, P., Sorokin, A., Ehrlich, S. D., Kulakauskas, S., et al. (2004). Complete sequence and comparative genome analysis of the dairy bacterium Streptococcus thermophilus. *Nat Biotechnol* 22, 1554–1558. doi:10.1038/nbt1034.

Bourgogne, A., Garsin, D. A., Qin, X., Singh, K. V., Sillanpaa, J., Yerrapragada, S., et al. (2008). Large scale variation in Enterococcus faecalis illustrated by the genome analysis of strain OG1RF. *Genome Biol* 9, R110. doi:10.1186/gb-2008-9-7-r110.

Brede, D. A., Snipen, L. G., Ussery, D. W., Nederbragt, A. J., and Nes, I. F. (2011). Complete Genome Sequence of the Commensal Enterococcus faecalis 62, Isolated from a Healthy Norwegian Infant. *Journal of Bacteriology* 193, 2377–2378. doi:10.1128/JB.00183-11.

Butler, R. R., Soomer-James, J. T. A., Frenette, M., and Pombert, J.-F. (2017). Complete Genome Sequences of Two Human Oral Microbiome Commensals, *Streptococcus salivarius* ATCC 25975 and *S. salivarius* ATCC 27945. *Genome Announc.* 5, e00536-17, /ga/5/24/e00536-17.atom. doi:10.1128/genomeA.00536-17.

Camelo-Castillo, A., Benitez-Paez, A., Belda-Ferre, P., Cabrera-Rubio, R., and Mira, A. (2014). Streptococcus dentisani sp. nov., a novel member of the mitis group. *INTERNATIONAL JOURNAL OF SYSTEMATIC AND EVOLUTIONARY MICROBIOLOGY* 64, 60–65. doi:10.1099/ijs.0.054098-0.

Chan, K.-G., Ng, K. T., Pang, Y. K., Chong, T. M., Kamarulzaman, A., Yin, W.-F., et al. (2015). Genome Anatomy of *Streptococcus parasanguinis* Strain C1A, Isolated from a Patient with Acute Exacerbation of Chronic Obstructive Pulmonary Disease, Reveals Unusual Genomic Features. *Genome Announc.* 3, e00541-15, /ga/3/3/e00541-15.atom. doi:10.1128/genomeA.00541-15.

Delorme, C., Bartholini, C., Luraschi, M., Pons, N., Loux, V., Almeida, M., et al. (2011a). Complete Genome Sequence of the Pigmented Streptococcus thermophilus Strain JIM8232. *Journal of Bacteriology* 193, 5581–5582. doi:10.1128/JB.05404-11.

Delorme, C., Guedon, E., Pons, N., Cruaud, C., Couloux, A., Loux, V., et al. (2011b). Complete Genome Sequence of the Clinical Streptococcus salivarius Strain CCHSS3. *Journal of Bacteriology* 193, 5041–5042. doi:10.1128/JB.05416-11.

Denapaite, D., Brückner, R., Nuhn, M., Reichmann, P., Henrich, B., Maurer, P., et al. (2010). The Genome of Streptococcus mitis B6 - What Is a Commensal? *PLoS ONE* 5, e9426. doi:10.1371/journal.pone.0009426.

Denapaite, D., Rieger, M., Köndgen, S., Brückner, R., Ochigava, I., Kappeler, P., et al. (2016). Highly Variable *Streptococcus oralis* Strains Are Common among Viridans Streptococci Isolated from Primates. *mSphere* 1, e00041-15, /msph/1/2/e00041-15.atom. doi:10.1128/mSphere.00041-15.

Diene, S. M., François, P., Zbinden, A., Entenza, J. M., and Resch, G. (2016). Comparative Genomics Analysis of Streptococcus tigurinus Strains Identifies Genetic Elements Specifically and Uniquely Present in Highly Virulent Strains. *PLoS ONE* 11, e0160554. doi:10.1371/journal.pone.0160554.

Doumith, M., Cazalet, C., Simoes, N., Frangeul, L., Jacquet, C., Kunst, F., et al. (2004). New Aspects Regarding Evolution and Virulence of Listeria monocytogenes Revealed by Comparative Genomics and DNA Arrays. *Infection and Immunity* 72, 1072–1083. doi:10.1128/IAI.72.2.1072-1083.2004.

Fritzenwanker, M., Kuenne, C., Billion, A., Hain, T., Zimmermann, K., Goesmann, A., et al. (2013). Complete Genome Sequence of the Probiotic Enterococcus faecalis Symbioflor 1 Clone DSM 16431. *Genome Announcements* 1, e00165-12, 1/1/e00165-12. doi:10.1128/genomeA.00165-12.

Geng, J., Chiu, C.-H., Tang, P., Chen, Y., Shieh, H.-R., Hu, S., et al. (2012). Complete Genome and Transcriptomes of Streptococcus parasanguinis FW213: Phylogenic Relations and Potential Virulence Mechanisms. *PLoS ONE* 7, e34769. doi:10.1371/journal.pone.0034769.

Geng, J., Huang, S.-C., Li, S., Hu, S., and Chen, Y.-Y. M. (2011). Complete Genome Sequence of the Ureolytic Streptococcus salivarius Strain 57.I. *Journal of Bacteriology* 193, 5596–5597. doi:10.1128/JB.05670-11.

Gilmour, M. W., Graham, M., Van Domselaar, G., Tyler, S., Kent, H., Trout-Yakel, K. M., et al. (2010). High-throughput genome sequencing of two Listeria monocytogenes clinical isolates during a large foodborne outbreak. *BMC Genomics* 11, 120. doi:10.1186/1471-2164-11-120.

Glaser, P., Rusniok, C., Buchrieser, C., Chevalier, F., Frangeul, L., Msadek, T., et al. (2002). Genome sequence of Streptococcus agalactiae, a pathogen causing invasive neonatal disease: Genome sequence of Streptococcus agalactiae. *Molecular Microbiology* 45, 1499–1513. doi:10.1046/j.1365-2958.2002.03126.x.

Guedon, E., Delorme, C., Pons, N., Cruaud, C., Loux, V., Couloux, A., et al. (2011). Complete Genome Sequence of the Commensal Streptococcus salivarius Strain JIM8777. *Journal of Bacteriology* 193, 5024–5025. doi:10.1128/JB.05390-11.

Hasegawa, N., Sekizuka, T., Sugi, Y., Kawakami, N., Ogasawara, Y., Kato, K., et al. (2017). Characterization of the Pathogenicity of Streptococcus intermedius TYG1620 Isolated from a Human Brain Abscess Based on the Complete Genome Sequence with Transcriptome Analysis and Transposon Mutagenesis in a Murine Subcutaneous Abscess Model. *Infection and Immunity* 85. doi:10.1128/IAI.00886-16.

Heng, N. C. K., Haji-Ishak, N. S., Kalyan, A., Wong, A. Y. C., Lovrić, M., Bridson, J. M., et al. (2011). Genome Sequence of the Bacteriocin-Producing Oral Probiotic Streptococcus salivarius Strain M18. *J. Bacteriol.* 193, 6402–6403. doi:10.1128/JB.06001-11.

Holden, M. T. G., Scott, A., Cherevach, I., Chillingworth, T., Churcher, C., Cronin, A., et al. (2007). Complete Genome of Acute Rheumatic Fever-Associated Serotype M5 Streptococcus pyogenes Strain Manfredo. *Journal of Bacteriology* 189, 1473–1477. doi:10.1128/JB.01227-06.

Hoskins, J., Alborn, W. E., Arnold, J., Blaszczak, L. C., Burgett, S., DeHoff, B. S., et al. (2001). Genome of the Bacterium Streptococcus pneumoniae Strain R6. *Journal of Bacteriology* 183, 5709–5717. doi:10.1128/JB.183.19.5709-5717.2001.

Kang, X., Ling, N., Sun, G., Zhou, Q., Zhang, L., and Sheng, Q. (2012). Complete Genome Sequence of Streptococcus thermophilus Strain MN-ZLW-002. *Journal of Bacteriology* 194, 4428–4429. doi:10.1128/JB.00740-12.

Kilian, M., Riley, D. R., Jensen, A., Brüggemann, H., and Tettelin, H. (2014). Parallel Evolution of Streptococcus pneumoniae and Streptococcus mitis to Pathogenic and Mutualistic Lifestyles. *mBio* 5, e01490-14. doi:10.1128/mBio.01490-14.

Kopit, L. M., Kim, E. B., Siezen, R. J., Harris, L. J., and Marco, M. L. (2014). Safety of the Surrogate Microorganism *Enterococcus faecium* NRRL B-2354 for Use in Thermal Process Validation. *Appl. Environ. Microbiol.* 80, 1899–1909. doi:10.1128/AEM.03859-13.

Lam, M. M. C., Seemann, T., Bulach, D. M., Gladman, S. L., Chen, H., Haring, V., et al. (2012). Comparative Analysis of the First Complete Enterococcus faecium Genome. *Journal of Bacteriology* 194, 2334–2341. doi:10.1128/JB.00259-12.

Lam, M. M., Seemann, T., Tobias, N. J., Chen, H., Haring, V., Moore, R. J., et al. (2013). Comparative analysis of the complete genome of an epidemic hospital sequence type 203 clone of vancomycin-resistant Enterococcus faecium. *BMC Genomics* 14, 595. doi:10.1186/1471-2164-14-595.

Lanie, J. A., Ng, W.-L., Kazmierczak, K. M., Andrzejewski, T. M., Davidsen, T. M., Wayne, K. J., et al. (2007). Genome Sequence of Avery’s Virulent Serotype 2 Strain D39 of Streptococcus pneumoniae and Comparison with That of Unencapsulated Laboratory Strain R6. *Journal of Bacteriology* 189, 38–51. doi:10.1128/JB.01148-06.

Liu, G., Zhang, W., and Lu, C. (2012). Complete Genome Sequence of Streptococcus agalactiae GD201008-001, Isolated in China from Tilapia with Meningoencephalitis. *J. Bacteriol.* 194, 6653–6653. doi:10.1128/JB.01788-12.

Makarova, K., Slesarev, A., Wolf, Y., Sorokin, A., Mirkin, B., Koonin, E., et al. (2006). Comparative genomics of the lactic acid bacteria. *Proceedings of the National Academy of Sciences* 103, 15611–15616. doi:10.1073/pnas.0607117103.

Maruyama, F., Kobata, M., Kurokawa, K., Nishida, K., Sakurai, A., Nakano, K., et al. (2009). Comparative genomic analyses of Streptococcus mutans provide insights into chromosomal shuffling and species-specific content. *BMC Genomics* 10, 358. doi:10.1186/1471-2164-10-358.

Nakagawa, I. (2003). Genome Sequence of an M3 Strain of Streptococcus pyogenes Reveals a Large-Scale Genomic Rearrangement in Invasive Strains and New Insights into Phage Evolution. *Genome Research* 13, 1042–1055. doi:10.1101/gr.1096703.

Natarajan, P., and Parani, M. (2015). First Complete Genome Sequence of a Probiotic Enterococcus faecium Strain T-110 and Its Comparative Genome Analysis with Pathogenic and Non-pathogenic Enterococcus faecium Genomes. *Journal of Genetics and Genomics* 42, 43–46. doi:10.1016/j.jgg.2014.07.002.

Nelson, K. E. (2004). Whole genome comparisons of serotype 4b and 1/2a strains of the food-borne pathogen Listeria monocytogenes reveal new insights into the core genome components of this species. *Nucleic Acids Research* 32, 2386–2395. doi:10.1093/nar/gkh562.

Olson, A. B., Kent, H., Sibley, C. D., Grinwis, M. E., Mabon, P., Ouellette, C., et al. (2013). Phylogenetic relationship and virulence inference of Streptococcus Anginosus Group: curated annotation and whole-genome comparative analysis support distinct species designation. *BMC Genomics* 14, 895. doi:10.1186/1471-2164-14-895.

Palma, T. H., Harth-Chú, E. N., Scott, J., Stipp, R. N., Boisvert, H., Salomão, M. F., et al. (2016). Oral cavities of healthy infants harbour high proportions of Streptococcus salivarius strains with phenotypic and genotypic resistance to multiple classes of antibiotics. *Journal of Medical Microbiology* 65, 1456–1464. doi:10.1099/jmm.0.000377.

Paulsen, I. T. (2003). Role of Mobile DNA in the Evolution of Vancomycin-Resistant Enterococcus faecalis. *Science* 299, 2071–2074. doi:10.1126/science.1080613.

Pereira, U. de P., Rodrigues dos Santos, A., Hassan, S. S., Aburjaile, F. F., Soares, S. de C., Ramos, R. T. J., et al. (2013). Complete genome sequence of Streptococcus agalactiae strain SA20-06, a fish pathogen associated to meningoencephalitis outbreaks. *Stand. Genomic Sci.* 8, 188–197. doi:10.4056/sigs.3687314.

Petrosyan, V., Holder, M., Ajami, N. J., Petrosino, J. F., Sahasrabhojane, P., Thompson, E. J., et al. (2016). Complete Genome Sequence of *Streptococcus mitis* Strain SVGS_061 Isolated from a Neutropenic Patient with Viridans Group Streptococcal Shock Syndrome. *Genome Announc.* 4, e00259-16, /ga/4/2/e00259-16.atom. doi:10.1128/genomeA.00259-16.

Qin, X., Galloway-Peña, J. R., Sillanpaa, J., Roh, J., Nallapareddy, S. R., Chowdhury, S., et al. (2012). Complete genome sequence of Enterococcus faecium strain TX16 and comparative genomic analysis of Enterococcus faecium genomes. *BMC Microbiol* 12, 135. doi:10.1186/1471-2180-12-135.

Rahman, M., Nguyen, S. V., McCullor, K. A., King, C. J., Jorgensen, J. H., and McShan, W. M. (2015). Complete Genome Sequence of *Streptococcus anginosus* J4211, a Clinical Isolate. *Genome Announc.* 3, e01440-15, /ga/3/6/e01440-15.atom. doi:10.1128/genomeA.01440-15.

Rasmussen, L. H., Dargis, R., Højholt, K., Christensen, J. J., Skovgaard, O., Justesen, U. S., et al. (2016). Whole genome sequencing as a tool for phylogenetic analysis of clinical strains of Mitis group streptococci. *Eur J Clin Microbiol Infect Dis* 35, 1615–1625. doi:10.1007/s10096-016-2700-2.

Reichmann, P., Nuhn, M., Denapaite, D., Bruckner, R., Henrich, B., Maurer, P., et al. (2011). Genome of Streptococcus oralis Strain Uo5. *Journal of Bacteriology* 193, 2888–2889. doi:10.1128/JB.00321-11.

Sabharwal, A., Liao, Y.-C., Lin, H.-H., Haase, E. M., and Scannapieco, F. A. (2015). Draft Genome Sequences of 18 Oral Streptococcus Strains That Encode Amylase-Binding Proteins: TABLE 1. *Genome Announc.* 3, e00510-15, /ga/3/3/e00510-15.atom. doi:10.1128/genomeA.00510-15.

Schijffelen, M. J., Boel, C. E., van Strijp, J. A., and Fluit, A. C. (2010). Whole genome analysis of a livestock-associated methicillin-resistant Staphylococcus aureus ST398 isolate from a case of human endocarditis. *BMC Genomics* 11, 376. doi:10.1186/1471-2164-11-376.

Shi, Y., Chen, Y., Li, Z., Yang, L., Chen, W., and Mu, Z. (2015). Complete Genome Sequence of *Streptococcus thermophilus* MN-BM-A02, a Rare Strain with a High Acid-Producing Rate and Low Post-Acidification Ability. *Genome Announc.* 3, e00979-15, /ga/3/5/e00979-15.atom. doi:10.1128/genomeA.00979-15.

Smoot, J. C., Barbian, K. D., Van Gompel, J. J., Smoot, L. M., Chaussee, M. S., Sylva, G. L., et al. (2002). Genome sequence and comparative microarray analysis of serotype M18 group A Streptococcus strains associated with acute rheumatic fever outbreaks. *Proceedings of the National Academy of Sciences* 99, 4668–4673. doi:10.1073/pnas.062526099.

Srinivasan, V., Metcalf, B. J., Knipe, K. M., Ouattara, M., McGee, L., Shewmaker, P. L., et al. (2014). vanG Element Insertions within a Conserved Chromosomal Site Conferring Vancomycin Resistance to Streptococcus agalactiae and Streptococcus anginosus. *mBio* 5, e01386-14. doi:10.1128/mBio.01386-14.

Steele, C. L., Donaldson, J. R., Paul, D., Banes, M. M., Arick, T., Bridges, S. M., et al. (2011). Genome Sequence of Lineage III Listeria monocytogenes Strain HCC23. *Journal of Bacteriology* 193, 3679–3680. doi:10.1128/JB.05236-11.

Sun, Z., Chen, X., Wang, J., Zhao, W., Shao, Y., Wu, L., et al. (2011). Complete Genome Sequence of Streptococcus thermophilus Strain ND03. *Journal of Bacteriology* 193, 793–794. doi:10.1128/JB.01374-10.

Tettelin, H. (2001). Complete Genome Sequence of a Virulent Isolate of Streptococcus pneumoniae. *Science* 293, 498–506. doi:10.1126/science.1061217.

Tettelin, H., Masignani, V., Cieslewicz, M. J., Donati, C., Medini, D., Ward, N. L., et al. (2005). Genome analysis of multiple pathogenic isolates of Streptococcus agalactiae: Implications for the microbial “pan-genome.” *Proceedings of the National Academy of Sciences* 102, 13950–13955. doi:10.1073/pnas.0506758102.

Tettelin, H., Masignani, V., Cieslewicz, M. J., Eisen, J. A., Peterson, S., Wessels, M. R., et al. (2002). Complete genome sequence and comparative genomic analysis of an emerging human pathogen, serotype V Streptococcus agalactiae. *Proceedings of the National Academy of Sciences* 99, 12391–12396. doi:10.1073/pnas.182380799.

Tong, H., Shang, N., Liu, L., Wang, X., Cai, J., and Dong, X. (2013). Complete Genome Sequence of an Oral Commensal, Streptococcus oligofermentans Strain AS 1.3089. *Genome Announc.* 1. doi:10.1128/genomeA.00353-13.

Treu, L., Vendramin, V., Bovo, B., Campanaro, S., Corich, V., and Giacomini, A. (2014a). Genome Sequences of Four Italian Streptococcus thermophilus Strains of Dairy Origin. *Genome Announcements* 2, e00126-14, 2/2/e00126-14. doi:10.1128/genomeA.00126-14.

Treu, L., Vendramin, V., Bovo, B., Campanaro, S., Corich, V., and Giacomini, A. (2014b). Genome Sequences of Streptococcus thermophilus Strains MTH17CL396 and M17PTZA496 from Fontina, an Italian PDO Cheese. *Genome Announcements* 2, e00067-14, 2/1/e00067-14. doi:10.1128/genomeA.00067-14.

Treu, L., Vendramin, V., Bovo, B., Campanaro, S., Corich, V., and Giacomini, A. (2014c). Whole-Genome Sequences of Streptococcus thermophilus Strains TH1435 and TH1436, Isolated from Raw Goat Milk. *Genome Announcements* 2, e01129-13, 2/1/e01129-13. doi:10.1128/genomeA.01129-13.

Van den Bogert, B., Boekhorst, J., Herrmann, R., Smid, E. J., Zoetendal, E. G., and Kleerebezem, M. (2013). Comparative Genomics Analysis of Streptococcus Isolates from the Human Small Intestine Reveals their Adaptation to a Highly Dynamic Ecosystem. *PLoS ONE* 8, e83418. doi:10.1371/journal.pone.0083418.

Wu, Q., Tun, H. M., Leung, F. C.-C., and Shah, N. P. (2015). Genomic insights into high exopolysaccharide-producing dairy starter bacterium Streptococcus thermophilus ASCC 1275. *Sci Rep* 4, 4974. doi:10.1038/srep04974.

Xu, P., Alves, J. M., Kitten, T., Brown, A., Chen, Z., Ozaki, L. S., et al. (2007). Genome of the Opportunistic Pathogen Streptococcus sanguinis. *Journal of Bacteriology* 189, 3166–3175. doi:10.1128/JB.01808-06.

Zheng, W., Tan, T. K., Paterson, I. C., Mutha, N. V. R., Siow, C. C., Tan, S. Y., et al. (2016). StreptoBase: An Oral Streptococcus mitis Group Genomic Resource and Analysis Platform. *PLoS ONE* 11, e0151908. doi:10.1371/journal.pone.0151908.

Zischka, M., Kuenne, C., Blom, J., Dabrowski, P. W., Linke, B., Hain, T., et al. (2012). Complete Genome Sequence of the Porcine Isolate Enterococcus faecalis D32. *Journal of Bacteriology* 194, 5490–5491. doi:10.1128/JB.01298-12.

Zou, Y., Xue, W., Luo, G., Deng, Z., Qin, P., Guo, R., et al. (2019). 1,520 reference genomes from cultivated human gut bacteria enable functional microbiome analyses. *Nat Biotechnol* 37, 179–185. doi:10.1038/s41587-018-0008-8.
